# Improved survival for patients diagnosed with chronic lymphocytic leukemia in the era of chemo-immunotherapy: a Danish population-based study of 10455 patients

**DOI:** 10.1038/bcj.2016.105

**Published:** 2016-11-11

**Authors:** C da Cunha-Bang, J Simonsen, K Rostgaard, C Geisler, H Hjalgrim, C U Niemann

**Affiliations:** 1 Department of Hematology, Rigshospitalet, Copenhagen University Hospital, Copenhagen, Denmark; 2 Department of Epidemiology Research, Statens Serum Institut – SSI, Copenhagen, Denmark

## Abstract

The treatment of chronic lymphocytic leukemia (CLL) is in rapid transition, and during recent decades both combination chemotherapy and immunotherapy have been introduced. To evaluate the effects of this development, we identified all CLL patients registered in the nation-wide Danish Cancer Register between 1978 and 2013. We identified 10 455 CLL patients and 508 995 CLL-free control persons from the general population. Compared with the latter, the relative mortality rate between CLL patients and their controls decreased from 3.4 (95% CI 3.2–3.6) to 1.9 (95% CI 1.7–2.1) for patients diagnosed in 1978–1984 and 2006–2013, respectively. The improved survival corresponded to a decreasing risk of death from malignant hematological diseases, whereas the risk of death from infections was stable during the study period. These population-based data substantiate the improved survival for patients treated with chemo-immunotherapy demonstrated in clinical studies.

## Introduction

Chronic lymphocytic leukemia (CLL) is the most common type of leukemia in adults with an estimated annual 15 000 new cases and 4500 deaths in the USA.^1^ CLL is a biologically and clinically heterogeneous disease and prognosis varies by age at diagnosis, comorbidity, clinical stage and CLL-specific molecular markers.^2, 3, 4, 5^

**Figure 1:**
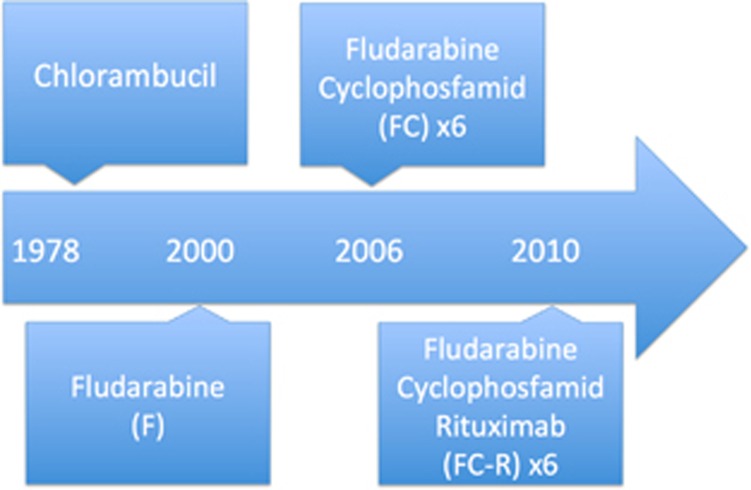
Overview of the guidelines for the management of CLL during the study period. Based on published studies with implications on the treatment in Denmark, national guidelines and market authorization for specific drugs.^6, 7, 10^

Treatment for CLL is evolving rapidly from chlorambucil over initially single-agent fludarabine (F) in 2000,^6^ then F combined with cyclophosphamide (FC) in 2006^7, 8, 9^ to FC combined with rituximab (FCR) in 2010 (Figure 1).^10^ Also, for patients with significant comorbidities the addition of CD20-targeting antibodies to chlorambucil has improved overall survival.^11^ Most recently, the introduction of BCR-targeted treatment (Ibrutinib and Idelalisib) for TP53 aberration-positive disease has vastly improved the outcome for patients with high-risk CLL.^12, 13^ Finally, the combinations of kinase inhibitors with chemo-immunotherapy have been shown to be superior to chemo-immunotherapy alone.^14, 15^

While the advantages of these new treatment modalities have been convincingly demonstrated in clinical trials, little is known about how they may have changed the survival of CLL patients in general.^16^ Specifically, participants in clinical trials may not always be representative of the general CLL patient population. In particular, a median age of 70 years at diagnosis implies that a substantial proportion of CLL patients are ineligible for inclusion in clinical trials. Consequently, trial results may not be generalizable to this large segment of the CLL patients.^17, 18^ In addition, clinical trials also do not include patients incidentally diagnosed with asymptomatic CLL, the number of which has increased in recent decades in conjunction to technical advances in diagnosis.^19, 20^

Despite the importance, information on the outcome of CLL outside clinical trials is barely available in the literature.^16, 17, 19, 21, 22^ In particular, the effects of chemo-immunotherapy as FCR, which is considered first-line treatment for the majority of fit patients, need further elucidation.^23, 24^

To remedy this shortcoming, we took advantage of the comprehensive nation-wide Danish Cancer Registry^25^ to assess changes in survival and causes of death (CODs) among CLL patients diagnosed in the period from 1978 to 2013. To account for secular trends in longevity in general, we compared the cohort of CLL patients with a matched cohort of individuals without CLL identified in the general Danish population.

## Materials and methods

### Study participants

We identified all patients registered with CLL (ICD10 code 91.1) between 1978 and 2013 (inclusive) in the nation-wide Danish Cancer Register.^25^ Using the personal identification number (PIN) unique to all Danish citizens since 2 April 1968 as key, we linked the cohort of CLL patients to the Danish Central Person Register (CPR), which continuously monitors the vital status of the Danish population.^26^ In the CPR, for each CLL patient we identified up to 50 individuals (comparison cohort; 92.9% of the CLL patients had 50 matched controls, 99.3% had more than 10 matched controls) from the general population, matched by sex, age (±1 year) and region of residence (municipality), who were alive and without CLL at the time of diagnosis of the index patient.

Information on underlying COD for deceased individuals in the two cohorts was ascertained from the nation-wide Danish Cause of Death Register^27^ through register linkage using the PIN as key. For the purpose of the present analyses, CODs were categorized into hematological/lymphatic malignancies, other malignancies, cardiovascular disease, cerebrovascular disease, infection and other and unknown CODs combined (see Supplementary Table S4 for a complete list of ICD8 and ICD10 codes used). In addition to underlying COD, we also assessed infection as contributory COD.

### Follow-up and statistical methods

All study participants were followed from the time of diagnosis/pseudodiagnosis until death, emigration, disappearance, or 31 December 2013, whichever came first.

Participant survival was calculated by Kaplan–Meier curves stratified by the calendar period of diagnosis (1978–1984, 1985–1991, 1992–1998, 1999–2005 and 2006–2013), age at diagnosis (younger than 65 years, 65–74 years, and 75 years and older) to explore changes in treatment and age-specific trends.

We used the Aalen-Johansen estimator, interpretable as a multidimensional generalization of the Kaplan–Meier estimator, to evaluate the risk of death from specific underlying causes.^28^

To quantify differences in overall and cause-specific mortality rates between age groups, genders and calendar periods of diagnosis we calculated hazard rate (HR) ratios with 95% confidence interval (95% CI) by Cox regression with time since diagnosis as underlying time scale. To overcome the extreme variation in mortality rates between patients and population controls by time since diagnosis, we used restricted cubic splines to model the time dependence.^29^

To reduce potential bias caused by earlier diagnosis of CLL in recent calendar periods, sensitivity analyses excluding the first year following diagnosis were performed. All analyses were performed using SAS version 9.4 (Cary, NC, USA).

The project was approved by The Danish Health and Medicines Authority and The Danish Data Protection Agency (j.nr. 2008-54-0472).

## Results and discussion

In total, 10 455 patients were registered with CLL in Denmark from 1978 to 2013 with 508 995 CLL-free individuals from the general population as matched controls. The majority of the patients were males (59%) and the median age at diagnosis was 72 years (Table 1).

**Figure 2:**
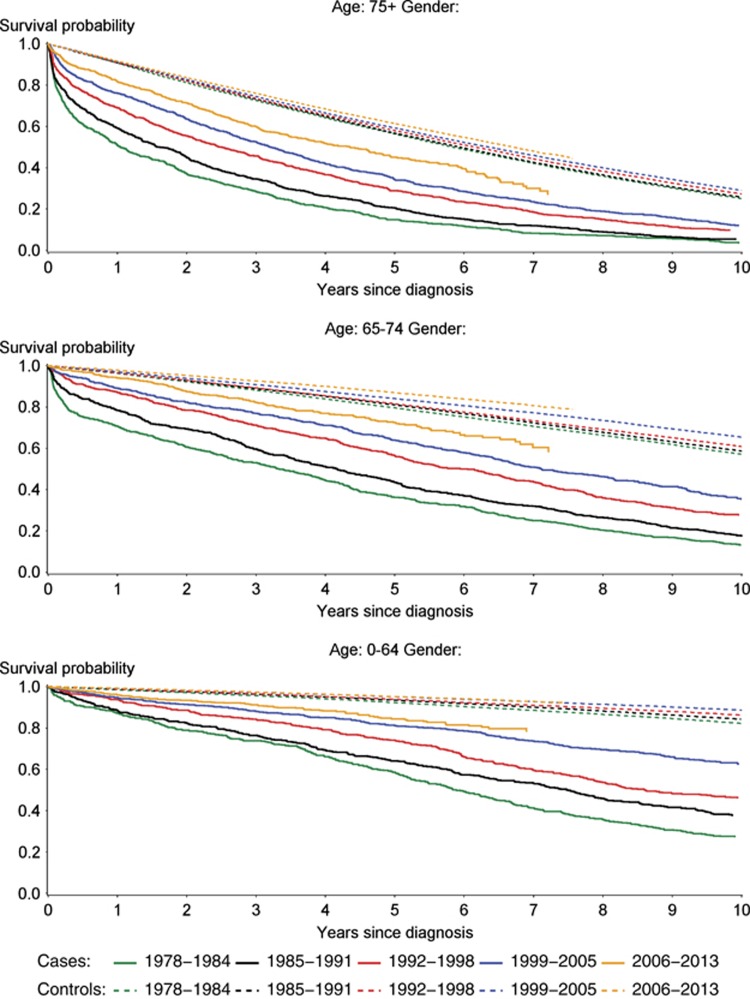
Survival from the time of CLL diagnosis. Survival probability for patients diagnosed with CLL (cases) and age, gender and region of residence matched background population (controls). Patients were categorized according to calendar period (1978–1984, 1985–1991, 1992–1998, 1999–2005 and 2006–2013) and age (under 64 (<64), from 65 to 74 or over 75 (⩾75)) years at the time of diagnosis. Cases and controls are plotted using full and dotted lines, respectively.

Continuously improved survival coinciding with changes of treatment options (Figure 1) was demonstrated for patients diagnosed with CLL during the study period. This is illustrated in Figure 2 showing Kaplan–Meier plots for CLL patients and matched controls. Correspondingly, the estimated 5-year overall survival increased monotonously across all age groups and in both sexes during the study period (Table 1). The improved survival during the study period could be demonstrated for both sexes and also for the youngest and oldest part of patients (less than 55 years or above 84 years of age) as seen from Kaplan–Meier curves in Supplementary Figure S1. Only compared with the earliest calendar periods, a slight increase in the incidence of CLL was seen, whereas the incidence of CLL was stable for the last three periods (Supplementary Table S3).

The Kaplan–Meier plots also suggested increasing longevity for the matched population cohort, albeit less pronounced than for the CLL patients (Figure 2).

When the CLL patients and the population sample were compared, a uniform decrease in mortality HR was apparent (HR_1978–1984, >365 days from diagnosis_=3.4 (3.2–3.6); HR_2006–2013, >365 days from diagnosis_ =1.9 (1.7–2.1)) (Table 2).

**Figure 3:**
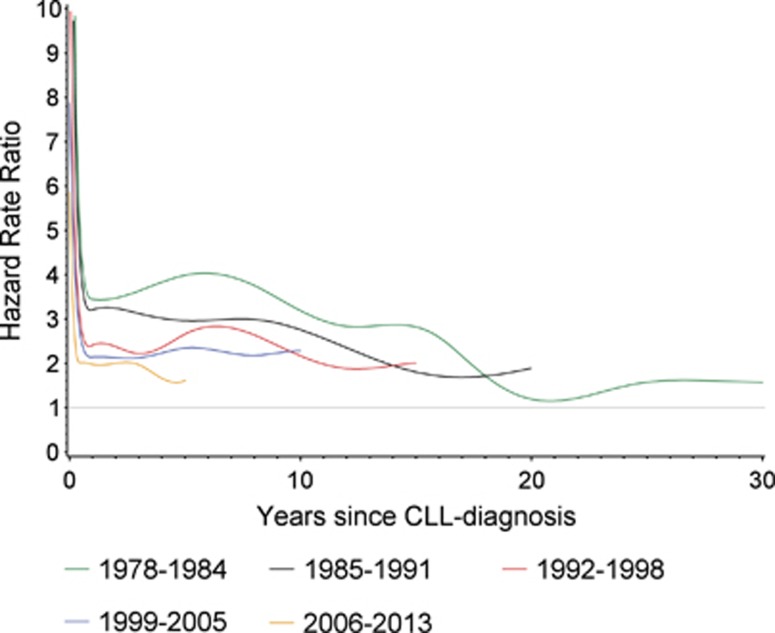
Mortality rate ratios according to calendar period of CLL diagnosis. The hazard rate ratio for death for patients diagnosed with CLL versus age, region of residence and gender matched background population. Patients were categorized according to calendar period (1978–1984, 1985–1991, 1992–1998, 1999–2005 and 2006–2013) of diagnosis.

Regardless of calendar period of diagnosis, the HR described a similar pattern by time since diagnosis. Specifically, HR decreased rapidly from extreme values within the first year after diagnosis and thereafter continued to decline at a slower pace (Table 2 and Figure 3). Of note, the decrease in mortality HR over calendar time of diagnosis applied across all periods of time since diagnosis. The successive decrease in mortality HR for each calendar period of diagnosis could be demonstrated for all age groups although some variation was seen (Table 2).

**Figure 4:**
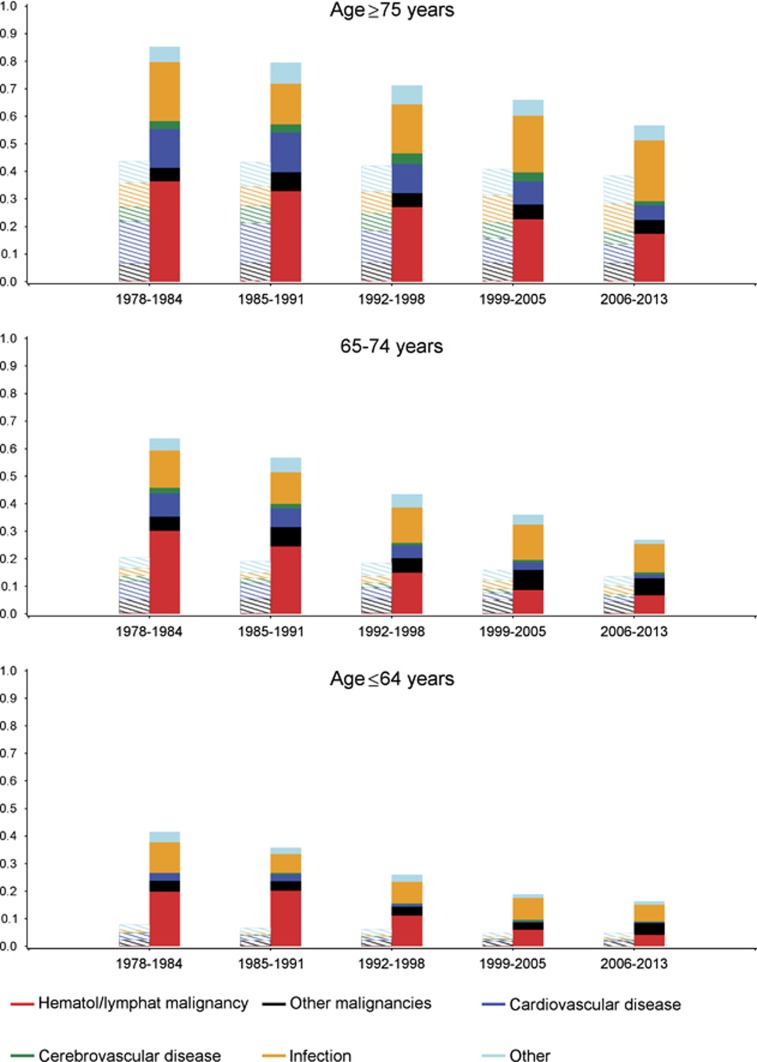
Cause of death according to calendar period and age at the time of CLL diagnosis. Cause-specific mortality for patients diagnosed with CLL versus age, gender and region of residence matched background population. Patients were categorized according to calendar period of diagnosis (1978–1984, 1985–1991, 1992–1998, 1999–2005 and 2006–2013) and age (under 64 (<64), from 65 to 74 and above 75 (⩾75)) at the time of diagnosis. Cause of death was categorized as hematological/lymphatic malignancy, other malignancies, cardiovascular disease, cerebrovascular disease, infection and other. Only underlying cause of death was included, except for infection where both contributory and underlying cause of death were included. Thus, infection as contributory or underlying cause of death overrules other underlying causes of death, each patient/control person only counted once. CLL patients are plotted using full color bars, matched background control using dashed bars.

The reduced overall mortality of the CLL patients primarily resulted from decreased risk of death from hematological/lymphoid malignancies as seen from cause-specific mortality rates based on the Aalen-Johansen estimator (Figure 4 and Supplementary Table S1). Risk of death from cardiovascular disease decreased over time for both CLL patients and matched controls, whereas the risk of death from other (non-hematological) malignancies was stable. The risk of death from infections was stable over time except for patients >75 years, where a slight increase was observed in 2006–2013 (Figure 4 and Supplementary Table S2).

Based on data from the Danish national registers we demonstrated a marked improvement in overall survival for CLL patients diagnosed in the period 1978-2013. This development was apparent in both sexes and across all age groups and coincided with the introduction of new treatments for CLL.^2, 30, 31, 32^

Our observations add considerably to the previously published data on changes in survival for patients with CLL.^16, 19, 21, 22^ In a recent study based on the Swedish cancer register (1972–2003), significantly improved 5- and 10-year survival was seen for patients >50 years at the time of diagnosis, whereas the mortality had been stable for younger patients since the early 1980s.^16^ In contrast, we also observed improved survival for patients below 50 years of age at diagnosis in the present analyses. Most importantly, the current study is the first to include unselected patients from the period with combination chemo-immunotherapy as the standard of care for patients with CLL.^33^

In our analyses, the increased survival among CLL patients exceeded and therefore could not be explained by increasing longevity of the general Danish population. Accordingly, other factors need to be considered in explanation for the observed development. The composition of the CLL population has undoubtedly changed during the study period. Not least has the proportion of patients with incidental diagnosis of asymptomatic CLL in all likelihood increased over time, which may have contributed to the apparently improved survival due to lead-time bias.^19^

Presumably, the most important mechanisms underlying the improved prognosis for CLL patients are the introduction of new treatment regimens combined with advances in supportive care, including surveillance and treatment for infectious complications during the study period.^10, 34^ The suggested association between treatment changes and improved survival is supported by significant improvements in survival for both the youngest (<64 years) and intermediate (65–74 years) age groups of patients diagnosed in 2006–2013 as compared with the previous period. This coincides with the introduction of FCR as standard therapy for fit CLL patients. As such, our findings expand on the results of randomized trials in which FCR regimens were shown to improve overall and progression-free survival compared with FC alone.^10, 35^ Likewise, a shift from single-agent chlorambucil to F and later FC could explain the improvements in overall survival seen in the youngest (<64 years) and intermediate (65–74 years) age groups diagnosed in 1992–1998 as compared with 1985–1991. This is also consistent with a number of clinical trials showing improved progression-free survival with more intensified combination chemotherapy.^6, 36^

An improvement in survival was also observed for the oldest (>75 years) CLL patients diagnosed in 1999–2005 and 2006–2013, respectively. As this stratum of the CLL cohort represents patients with increased potential of toxicity and higher frequency of comorbidity,^37^ the introduction of semi-intensive chemotherapy like Bendamustine and reduced intensity FCR for this population probably underlies these observations.^38^ In addition, the use of CD20-targeting antibodies and a more aggressive treatment strategy, as recently shown in clinical trials, could also play a role.^39^ Based on the scarcity of clinical trials including elderly, frail patients, the data presented here on improved overall survival also for this patient group are noteworthy.

Finally, improvements in supportive care for patients with CLL most likely contributed substantially to the improved overall survival for all age groups. Here, improved managements of infections and autoimmune cytopenias along with better support for transfusion of blood products as well as immunoglobulin substitution may have had a significant impact.^40, 41^

To further assess the potential impact of improvements in supportive care, CLL-specific treatment and other causes, we analyzed changes in underlying cause of death over time. Significant morbidity and mortality due to infections caused by CLL itself and by treatment-related adverse events are reported in clinical trials and seen in daily clinical practice.^42^ Only controlled trials can show whether intensive chemo-immunotherapy put patients at greater risk of severe infectious complications. However, no changes in risk of death from infection as underlying cause or contributory cause was seen over time. Approximately 50% increased risk of death with infections as contributory or underlying cause was demonstrated compared with the matched controls in accordance with previous reports.^43^ These findings are reassuring for the introduction of more intensive treatment also for fragile patients with CLL and comorbidity in recent years. Previous reports showing increased risk of death from secondary malignancies for patients with CLL compared with the background population could not be confirmed in the present study.^44, 45^ A possible explanation for this discrepancy could be that patients diagnosed with CLL in our study have a shorter time at risk of dying from another malignancy, due to increased risk of death from CLL and no differentiation between deaths from CLL and other hematological malignancies. The clear decrease in deaths attributable to hematological malignancies (including CLL) emphasizes the overall impact of better treatment for CLL during the study period on survival.

Age remains an important predictor of survival in all calendar periods of diagnosis, both for patients with CLL and matched controls, reflecting the basic correlation for increased risk of comorbidity and decreased life expectancy with increasing age.^46^ Throughout the study period, male CLL patients had a worse survival outcome than female patients (Supplementary Figure S1). This observation corroborates several previous reports.^16, 47, 48^ It emphasizes an unmet need for treatment options improving the outcome further also for male patients with CLL, as seen from the overall survival for female patients in the youngest age group approaching that of matched controls, while survival for young male CLL patients is still lagging behind. Sex-specific differences in comorbidity and lifestyle may account for part of this; however, sex-specific factors for CLL itself remain to be investigated further.

The validity of our findings are supported by the use of population-based cohorts from a nation-wide register with a very high coverage.^25, 27^ The large number of patients, matched controls and end points yielded a high statistical power to detect even small changes in survival over time. However, the current study spans 35 years with major changes not only regarding therapy but also diagnostic criteria for CLL.^2^ The number of patients incidentally diagnosed with asymptomatic CLL (Stage A) has increased in recent decades.^19, 20^ This reflects that a higher proportion of patients at earlier calendar periods of diagnosis probably had more progressive stages of CLL at the time of diagnosis, thus had a worse outcome. Regarding COD: registration bias in the COD registry towards acknowledging CLL as a cause of death among patients diagnosed with CLL despite another disease being the primary cause of death could potentially bias the results. The lack of clinical data, treatment data and specific prognostic variables for individual patients are limitations to the current study that will be addressed by future studies based on the Danish National CLL database.^49, 50^

We provide the first population-based data demonstrating significant improvement in survival for patients with CLL in parallel with the introduction of chemo-immunotherapeutic regimens into clinical practice. This substantiates the reported improved survival for patients treated with chemo-immunotherapy in clinical trials, supporting the continued importance of chemo-immunotherapy for frontline treatment of CLL. The impact of novel targeted agents for CLL and outcome for specific patient groups outside clinical trials awaits further maturity of data from registries like the Danish National CLL database.^50^

## Figures and Tables

**Table 1:** Patients

*Year of diagnosis*			*1978–1984*	*1985–1991*	*1992–1998*	*1999–2005*	*2006–2013*	*Total*
Number of patients			1790	1967	2120	2433	2145	10455
Males (M)			1080	1181	1206	1419	1263	6149
Female (F)			710	786	914	1014	882	4306
Age, median (IQR)			72 (64*–*79)	73 (65*–*80)	73 (64*–*80)	72 (63*–*80)	71 (63*–*80)	72 (64–80)
								
*Age groups*								
<64			481 (27%)	490 (25%)	574 (27%)	730 (30%)	663 (31%)	2938 (28%)
65–74			650 (36%)	635 (32%)	649 (31%)	702 (29%)	646 (30%)	3282 (31%)
⩾75			659 (37%)	842 (43%)	897 (43%)	1001 (41%)	836 (39%)	4235 (41%)
								
*Estimated 5-year survival*								
<64								
M			54%	61%	72%	78%	81%	
F			66%	70%	78%	87%	91%	
65–74								
M			29%	37%	50%	60%	69%	
F			49%	55%	67%	71%	79%	
⩾75								
M			10%	19%	24%	30%	40%	
F			21%	23%	34%	39%	50%	

Patient characteristics of 10 455 patients diagnosed with CLL from 1978 to 2013. Patients were categorized according to calendar period (1978–1984, 1985–1991, 1992–1998, 1999–2005 and 2006–2013), age (under 64 (<64), from 65 to 74 or over 75 (⩾75)) and years at the time of diagnosis. Estimated 5-year survival is shown for each age category and male (M) and female (F) patients.

**Table 2:** Risk of death according to calendar period, time from and age at CLL diagnosis

*Calendar period*	*1978–1984*	*1985–1991*	*1992–1998*	*1999–2005*	*2006–2013*
*Time since diagnosis*					
<365 days	8.5 (7.8–9.3)	6.5 (5.9–7.1)	4.2 (3.7– 4.68)	3.4 (3.1–3.8)	2.6 (2.3–3.0)
1*–*4 years	3.6 (3.3–4.0)	3.1 (2.9–3.4)	2.3 (2.2–2.6)	2.3 (2.1–2.4)	1.9 (1.7–2.1)
5*–*10 years	3.7 (3.3–4.1)	3.0 (2.7–3.3)	2.5 (2.2–2.7)	2.3 (2.1–2.5)	1.9 (1.5–0.4)
10–15 years	3.1 (2.6–3.7)	2.3 (2.0–2.8)	2.2 (1.9–2.5)	1.9 (1.6–2.3)	NA
>15 years	1.7 (1.3–2.1)	1.9 (1.6–2.3)	2.0 (1.6–2.5)	NA	NA
Overall>365 days	3.4 (3.2–3.6)	2.8 (2.7–3.0)	2.3 (2.2–2.5)	2.3 (2.1–2.4)	1.9 (1.7–2.1)
Index	1.4 (1.3 *–*1.6)	1.2 (1.1–1.3)	Ref.	1.0 (0.9–1.0)	0.8 (0.7–0.9)
					
*Age at diagnosis*					
⩽54	7.7 (6.3–9.3)	6.4 (5.3–7.8)	7.8 (6.4–9.4)	5.9 (4.6–7.4)	2.8 (1.2–6.3)
55–64	4.8 (4.3–5.5)	3.6 (3.2–4.1)	3.9 (3.4–4.3)	3.2 (2.8–3.8)	3.1 (2.4–4.0)
65–74	3.0 (2.7–3.3)	2.9 (2.7–3.2)	2.3 (2.1–2.6)	2.4 (2.2–2.7)	2.3 (2.0–2.8)
75–84	2.6 (2.3–2.9)	2.2 (2.0–2.5)	1.9 (1.7–2.0)	1.9 (1.7–2.1)	1.8 (1.5–2.0)
⩾85	2.4 (1.8–3.2)	1.9 (1.6–2.4)	1.7 (1.4–2.0)	1.6 (1.3–1.8)	1.3 (1.0–1.6)

Rate ratios of death for CLL patients compared with age, region of residence and gender matched background population by calendar period of diagnosis (1978–1984, 1985–1991, 1992–1998, 1999–2005 and 2006-–013), time since and age at diagnosis.

Abbreviation: NA, not applicable.
