# Supplementary material for: Improved survival for patients diagnosed with chronic lymphocytic leukemia in the era of chemo-immunotherapy: a Danish population-based study of 10455 patients
Source: Blood Cancer J. 2016 Nov 11;6(11):e499–. doi: 10.1038/bcj.2016.105 (PMC5148052; doi:10.1038/bcj.2016.105)
Supplement: Supplemental Figure and Tables Legends [file bcj2016105x1.docx]

Supplementary Table 1.

Title: Cause of death according to calendar period and time since CLL diagnosis.

Legend: Rate ratio for cause-specific mortality for patients diagnosed with CLL versus age, gender and region of residence matched background population. Patients were categorized according to calendar period (1978-1984, 1985-1991, 1992-1998, 1999-2005 and 2006-2013) and time since diagnosis (<365 days, 2-4 years, 5-9 years, 10-15 years, >15 years and total > 365 days). Cause of death was categorized as; Hematological/lymphatic malignancy, Other malignancies, Cardiovascular disease, Cerebrovascular disease, Infection and Other.

Supplementary Table 2.

Title: Total number and number of infection related death in patients diagnosed with CLL and matched background population.

Legend: Total number (no) of deaths and infection related deaths in cases and controls according to calendar period (1978-1984, 1985-1991, 1992-1998, 1999-2005 and 2006-2013).

Supplementary Table 3.

Title: Rate ratio of risk of CLL according to calendar period.

Legend: Rate ratio of risk of CLL for calendar period 1985-1991, 1992-1998, 1999-2005 and 2006-2013 versus 1978-1984, respectively.

Supplementary Table 4.

Title: Cause of death ICD8 and ICD10 codes

Legend: ICD8 and ICD10 codes used to define cause of death

Supplementary Figure 1.

Title: Survival from time of CLL diagnosis according to age and gender.

Legend: Survival probability for patients diagnosed with CLL (cases) and age, gender and region of residence matched background population (controls). Patients were categorized according to calendar period of diagnosis (1978-1984, 1985-1991, 1992-1998, 1999-2005 and 2006-2013), gender (men and women) and age (from; 0-54, 55-64, 65-74, 75-84 or 85 and above (85+)) at the time of diagnosis. Cases and controls are plotted using full and dotted lines, respectively.
