# Supplementary material for: Improved survival for patients diagnosed with chronic lymphocytic leukemia in the era of chemo-immunotherapy: a Danish population-based study of 10455 patients
Source: Blood Cancer J. 2016 Nov 11;6(11):e499–. doi: 10.1038/bcj.2016.105 (PMC5148052; doi:10.1038/bcj.2016.105)
Supplement: Supplementary Table 1 [file bcj2016105x2.docx]

| Cause of death | Periode | >365 days | 2.-4. years | 5.-9. years | 10.-14. years | 15+years | Total (>365 days) |
| --- | --- | --- | --- | --- | --- | --- | --- |
| Hematol/lymphat malignancy | 1978-1984 | 364.4(267.1-497.2) | 144.9(117.6-178.6) | 171.7(128.4-229.6) | 116.4(71.53-189.3) | 40.39(23.01-70.92) | 138.2(118.6-161.0) |
|  | 1985-1991 | 234.6(178.9-307.5) | 105.0(87.18-126.5) | 111.4(86.62-143.3) | 93.61(60.87-143.9) | 41.97(25.74-68.42) | 99.40(86.80-113.8) |
|  | 1992-1998 | 132.8(103.2-170.9) | 70.85(60.03-83.62) | 104.9(83.93-131.1) | 67.19(48.41-93.25) | 33.26(13.78-80.28) | 78.66(69.67-88.82) |
|  | 1999-2005 | 106.6(82.84-137.2) | 60.36(51.47-70.77) | 77.81(62.19-97.34) | 44.33(22.89-85.86) | NA | 64.94(57.20-73.72) |
|  | 2006-2013 | 84.16(62.27-113.7) | 44.26(35.01-55.97) | 19.46( 4.83-78.49) | NA | NA | 43.19(34.29-54.41) |
|  |  |  |  |  |  |  |  |
| Other malignancies | 1978-1984 | 3.99( 3.02- 5.26) | 0.87( 0.62- 1.22) | 1.52( 1.08- 2.14) | 1.27( 0.71- 2.26) | 1.34( 0.70- 2.54) | 1.15( 0.93- 1.42) |
|  | 1985-1991 | 3.00( 2.29- 3.92) | 1.44( 1.13- 1.84) | 1.20( 0.87- 1.66) | 1.32( 0.82- 2.12) | 1.08( 0.63- 1.86) | 1.31( 1.11- 1.56) |
|  | 1992-1998 | 2.07( 1.52- 2.81) | 1.06( 0.83- 1.35) | 1.37( 1.06- 1.77) | 1.17( 0.80- 1.70) | 1.22( 0.49- 3.00) | 1.18( 1.01- 1.39) |
|  | 1999-2005 | 1.75( 1.27- 2.40) | 1.25( 1.02- 1.54) | 1.34( 1.03- 1.74) | 1.14( 0.51- 2.59) | NA | 1.28( 1.09- 1.50) |
|  | 2006-2013 | 1.75( 1.24- 2.46) | 1.30( 0.98- 1.72) | 0.58( 0.08- 4.17) | NA | NA | 1.27( 0.96- 1.67) |
|  |  |  |  |  |  |  |  |
| Cardiovascular disease | 1978-1984 | 3.30( 2.65- 4.12) | 1.38( 1.11- 1.72) | 0.97( 0.70- 1.35) | 1.24( 0.79- 1.95) | 0.86( 0.47- 1.58) | 1.19( 1.01- 1.40) |
|  | 1985-1991 | 2.79( 2.23- 3.51) | 1.36( 1.11- 1.67) | 1.18( 0.88- 1.58) | 0.78( 0.47- 1.31) | 1.02( 0.57- 1.82) | 1.21( 1.04- 1.41) |
|  | 1992-1998 | 1.79( 1.35- 2.37) | 1.25( 1.02- 1.53) | 0.70( 0.51- 0.97) | 0.95( 0.62- 1.44) | 1.81( 0.77- 4.23) | 1.03( 0.88- 1.21) |
|  | 1999-2005 | 1.77( 1.32- 2.39) | 1.15( 0.92- 1.44) | 0.66( 0.44- 0.98) | 1.47( 0.53- 4.04) | NA | 0.99( 0.82- 1.20) |
|  | 2006-2013 | 0.71( 0.38- 1.32) | 1.11( 0.79- 1.57) | 1.12( 0.15- 8.25) | NA | NA | 1.11( 0.79- 1.57) |
|  |  |  |  |  |  |  |  |
| Cerebrovascular disease | 1978-1984 | 1.15( 0.63- 2.10) | 1.18( 0.78- 1.79) | 1.07( 0.63- 1.83) | 1.16( 0.58- 2.30) | 0.93( 0.42- 2.04) | 1.11( 0.84- 1.47) |
|  | 1985-1991 | 1.84( 1.19- 2.85) | 0.63( 0.40- 0.99) | 0.94( 0.61- 1.46) | 0.16( 0.04- 0.65) | 1.06( 0.57- 1.99) | 0.70( 0.53- 0.93) |
|  | 1992-1998 | 1.11( 0.67- 1.82) | 0.78( 0.56- 1.10) | 0.74( 0.50- 1.09) | 0.52( 0.28- 0.99) | 0.61( 0.15- 2.52) | 0.72( 0.57- 0.90) |
|  | 1999-2005 | 1.00( 0.62- 1.60) | 0.65( 0.46- 0.93) | 0.53( 0.32- 0.88) | 1.65( 0.59- 4.62) | NA | 0.64( 0.48- 0.84) |
|  | 2006-2013 | 0.72( 0.36- 1.44) | 0.47( 0.25- 0.88) | NA | NA | NA | 0.46( 0.24- 0.85) |
|  |  |  |  |  |  |  |  |
| Infection | 1978-1984 | 6.65( 1.99-22.23) | 5.02( 1.98-12.69) | NA | 3.98( 0.48-32.82) | 1.85( 0.13-25.65) | 3.02( 1.35- 6.76) |
|  | 1985-1991 | 2.60( 0.35-19.50) | 3.61( 1.30-10.02) | NA | 2.92( 0.67-12.78) | NA | 1.88( 0.82- 4.33) |
|  | 1992-1998 | 1.88( 0.25-13.85) | 1.91( 0.60- 6.08) | 2.50( 0.99- 6.29) | 0.53( 0.07- 3.87) | NA | 1.45( 0.74- 2.85) |
|  | 1999-2005 | 2.74( 0.84- 8.99) | 0.67( 0.21- 2.09) | 0.62( 0.15- 2.51) | 2.13( 0.28-16.49) | NA | 0.73( 0.32- 1.64) |
|  | 2006-2013 | 2.43( 0.76- 7.77) | 1.34( 0.42- 4.24) | NA | NA | NA | 1.28( 0.41- 4.06) |
|  |  |  |  |  |  |  |  |
| Other | 1978-1984 | 3.73( 2.90- 4.81) | 1.24( 0.94- 1.63) | 1.18( 0.82- 1.69) | 1.58( 1.01- 2.46) | 0.74( 0.42- 1.30) | 1.19( 0.98- 1.43) |
|  | 1985-1991 | 2.42( 1.84- 3.18) | 1.21( 0.96- 1.53) | 1.23( 0.94- 1.62) | 1.49( 1.06- 2.09) | 1.28( 0.89- 1.82) | 1.27( 1.10- 1.47) |
|  | 1992-1998 | 1.71( 1.29- 2.26) | 0.99( 0.80- 1.22) | 1.07( 0.84- 1.35) | 1.19( 0.90- 1.58) | 1.81( 1.33- 2.45) | 1.14( 1.01- 1.29) |
|  | 1999-2005 | 1.01( 0.73- 1.42) | 0.91( 0.74- 1.10) | 1.32( 1.11- 1.55) | 1.52( 1.13- 2.06) | NA | 1.16( 1.03- 1.30) |
|  | 2006-2013 | 1.07( 0.78- 1.48) | 1.36( 1.17- 1.58) | 1.98( 1.53- 2.55) | NA | NA | 1.49( 1.30- 1.69) |
