# Supplementary material for: Improved survival for patients diagnosed with chronic lymphocytic leukemia in the era of chemo-immunotherapy: a Danish population-based study of 10455 patients
Source: Blood Cancer J. 2016 Nov 11;6(11):e499–. doi: 10.1038/bcj.2016.105 (PMC5148052; doi:10.1038/bcj.2016.105)
Supplement: Supplementary Table 2 [file bcj2016105x3.docx]

|  |  | 1978-1984 | 1985-1991 | 1992-1998 | 1999-2005 | 2006-2013 |
| --- | --- | --- | --- | --- | --- | --- |
| **No. of deaths** | Cases | 1174 | 1203 | 1069 | 1048 | 646 |
| **Infection related** |  | 274 | 226 | 289 | 353 | 189 |
| **(%)** |  | 23.3% | 18.8% | 27.0% | 33.7% | 29.3% |
|  |  |  |  |  |  |  |
|  | Controls | 21685 | 24377 | 25635 | 26526 | 17738 |
|  |  | 3643 | 3351 | 4409 | 5912 | 3326 |
|  |  | 16.8% | 13.8% | 17.2% | 22.3% | 18.8% |
