# Supplementary material for: Improved survival for patients diagnosed with chronic lymphocytic leukemia in the era of chemo-immunotherapy: a Danish population-based study of 10455 patients
Source: Blood Cancer J. 2016 Nov 11;6(11):e499–. doi: 10.1038/bcj.2016.105 (PMC5148052; doi:10.1038/bcj.2016.105)
Supplement: Supplementary Table 3 [file bcj2016105x4.docx]

| **Years of diagnosis** | **Rate Ratio (95% Confidence Interval** |
| --- | --- |
| 1985-1991 vs 1978-1984 | 1.06 (0.99 – 1.13) |
| 1992-1998 vs. 1978-1984 | 1.08 (1.01, 1.16) |
| 1999-2005 vs. 1978-1984 | 1.19 (1.12, 1.27) |
| 2006-2013 vs. 1978-1984 | 1.13 (1.05, 1.20) |
