# Supplementary material for: Improved survival for patients diagnosed with chronic lymphocytic leukemia in the era of chemo-immunotherapy: a Danish population-based study of 10455 patients
Source: Blood Cancer J. 2016 Nov 11;6(11):e499–. doi: 10.1038/bcj.2016.105 (PMC5148052; doi:10.1038/bcj.2016.105)
Supplement: Supplementary Table 4 [file bcj2016105x5.docx]

| Cause of death | ICD8 | ICD10 |
| --- | --- | --- |
| Hematological/lymphatic malignancy | 200-209 | C81-C96, D45-D47 |
| Other malignancies | 140-199 | C00-C80 |
| Cardiovascular disease | 391–398, 400–404, 410–414, 420–429, 441, 450 | I01, I020, I020A, I05-­I09, I10, I11, I13, I20-­I28, I30-­I52, I700, I71, I720, ­I790, I791 |
| Cerebrovascular disease | 430-438, 442 | I60 - ­I69, I725, I726 |
| Infection | 000–134, 136, 320–324, 420-422, 460-466, 470–474, 480–486, 513, 567, 575, 590, 612 | A00–B99, G00–G09, I30, I310, I311, I32–I33, J00–J22, K81, K65, N70, N00–N03 |
| Other | All remaining | All remaining |
