# Supplementary figures and images for: Improved survival for patients diagnosed with chronic lymphocytic leukemia in the era of chemo-immunotherapy: a Danish population-based study of 10455 patients
Source: Blood Cancer J. 2016 Nov 11;6(11):e499–. doi: 10.1038/bcj.2016.105 (PMC5148052; doi:10.1038/bcj.2016.105)

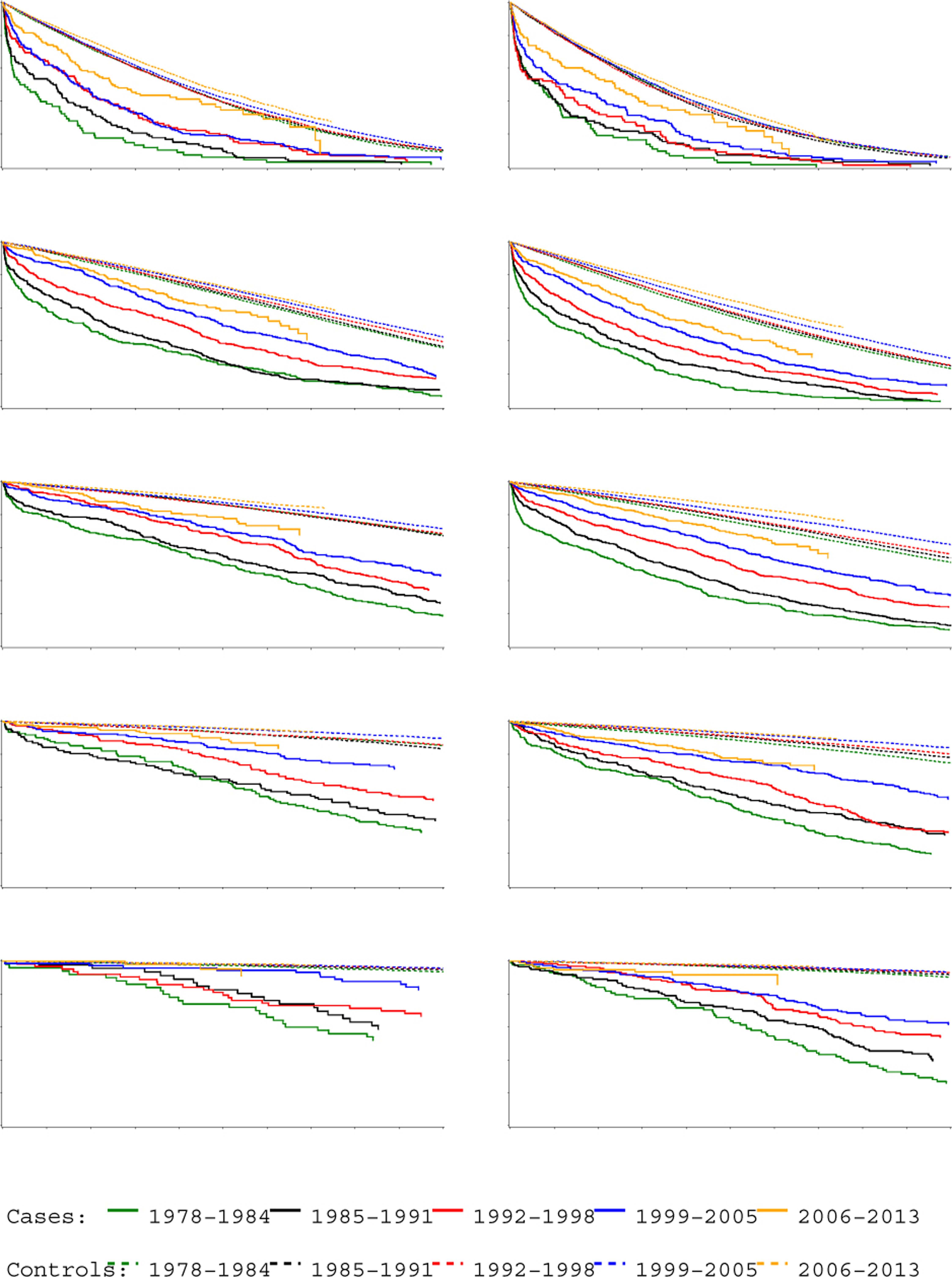

Supplement: Supplementary Figure 1 [file bcj2016105x6.tif]
